# Management of Fertigation in Horticultural Crops through Automation with Electrotensiometers: Effect on the Productivity of Water and Nutrients

**DOI:** 10.3390/s21010190

**Published:** 2020-12-30

**Authors:** Juana I. Contreras, Rafael Baeza, José G. López, Gema Cánovas, Francisca Alonso

**Affiliations:** Institute of Research and Training in Agriculture and Fishery (IFAPA), Junta of Andalucia, La Mojonera, 04630 Almería, Spain; rafaelj.baeza@juntadeandalucia.es (R.B.); jgabriel.lopez@juntadeandalucia.es (J.G.L.); gema.canovas@juntadeandalucia.es (G.C.); f.alonso@juntadeandalucia.es (F.A.)

**Keywords:** greenhouse, irrigation, activation threshold, water productivity, soil matric potential, drainage, biomass, harvest index

## Abstract

Water and nutrient requirements of horticultural crops are influenced by different factors such as: Type of crop, stage of development and production system. Although greenhouse horticultural crops are more efficient in the use of water and fertilizers compared to other production systems, it is necessary increase efficiency for which individualized fertigation strategies must be designed for each greenhouse. The automation of fertigation based on the level of soil moisture allows optimization of management. The objective of this work was to determine the influence of the activation command of fertigation with electrotensiometers and the characteristics of the greenhouse on the productivity of the crop and the efficiency of use of water and nutrients in a sweet pepper crop. The trial was developed in two greenhouses. Four treatments were studied, combination of who two-factor: Soil matric potential (SMP) (SMP_−10_: Automatic activation of irrigation to −10 kPa and SMP_−20_: Automatic activation of irrigation to −20 kPa) and greenhouse characteristics (G_1_ and G_2_). The nutritive solution applied was the same in all treatments. The yield and volume of water and nutrients applied were determined, calculating the productivity of the water (WP), as well as productivity the nutrients. The fertigation activation threshold of −10 kPa presented the best results, increasing the yield and conserving WP and nutrient productivity with respect to −20 kPa in both greenhouses. The automation of irrigation with electrotensiometers allowed the application of different volume of fertigation demanded by the crop in each greenhouse, equalizing the WP and nutrient productivity without producing drainage. The pepper crop in the greenhouse G_1_ presented greater vegetative development, higher yield and demanded a greater volume of fertigation than G_2_ regardless of the activation threshold. This was due to the fact that the soil matric potential after irrigation in greenhouse G_1_ was closer to zero, being able to conclude that not only the soil matric potential threshold of irrigation activation has an influence on crop, but also the potential registered after irrigation. Soil matric potentials closer to zero are more productive.

## 1. Introduction

In the current climate change scenario, the availability of water for human activities is expected to decrease [1]. Of these activities, agriculture is the one that consumes the most water, with irrigated agriculture accounting for 70% of freshwater extraction, and this percentage can reach 90% in some regions. Of the total water used, about 20% comes from groundwater sources (renewable or not), and this proportion is increasing rapidly, especially in dry areas [2]. Given this situation, with limited water resources, the use of drip irrigation systems is increasing throughout the world. This irrigation system can provide water to the plant frequently and directly in the root zone of the plant [3], being able to achieve a very high irrigation efficiency. Not only is the irrigation system important, irrigation management must also be efficient to minimize the possible environmental impact derived from it, as well as promote the sustainable use of resources [4,5]. In cultivation systems where drip irrigation systems are used, the use of fertigation for the supply of nutrients has become widespread, so in these systems soil is not irrigated, it is fertigated.

Horticultural crops have different fertigation needs depending on factors such as: Type of crop, stage of development and production system. Despite of the fact that greenhouse horticultural crops are very efficient in the use of water and fertilizers compared to other production systems, it is necessary to increase efficiency, for which individual fertigation strategies must be designed for each crop and each situation.

The water and nutritional needs of greenhouse horticultural crops have been extensively studied. The works that determine the water needs are mainly focused on the predictive calculation of crop evapotranspiration (ETc) with average climatic data or real-time data [6,7,8]. However, for the same crop, the different varieties show different vegetative development (some are more vegetative than others). Furthermore, the growing conditions of the crop can affect the vegetative development of the crop. For this reason, the most recent research has been directed towards the dynamic determination of the crop coefficient (K_c_) in situ, using photo digitization systems that monitor plant growth [9], and towards the use of humidity sensors of the soil that allow irrigation on demand [4].

Regarding fertigation, most of the research focuses on determining standard nutrient solutions for each crop and stage [10], as well as determining the extraction and absorption curves of the different crops [11,12,13,14]. As in irrigation, the different vegetative development generates, for the same crop, depending on the variety and growing conditions, different nutritional needs. Hence, the most recent investigations focus on the dynamic determination of these needs in situ through: Use of optical sensors to determine the nitrogen content in crops [15,16], use of systems for monitoring the level of nutrients in the soil solution [13,17,18] and development and adaptation of different simulation models of nutrient requirements [19,20].

The cultivation system has a great influence on the consumption of water and fertilizers [20,21,22]. Within the greenhouse cultivation system there are several factors that determine and modify the consumption of water and fertilizers, since they modify the environmental conditions in which they develop, such as: Greenhouse structure, ventilation and type of soil. [23,24,25,26]. Not only do the structural factors have influence, but so do the cultivation techniques such as pruning, phytosanitary treatments, since they modify the index of leaf area and the biomass of the cultivation.

Since the late 1980s, great advances in electronics and information technologies have led to significant progress in the development, availability, and application of sensors for use in irrigation scheduling and automation. Electrotensiometers are soil matric potential (SMP) sensors for a continuous control of water application by a computer. The automation of irrigation using electrotensiometers can be a viable option at the farm level that, among other advantages, offers the possibility of watering according to the individual characteristics of greenhouses and crops and can provide a more precise adjustment of the irrigation frequency to the crop needs at every moment of the cycle, minimizing losses due to drainage to deeper layers. In addition, limiting drainage, also is preventing nutrient leaching that can cause contamination of aquifers. However, for automatic irrigation control based on a value of the soil matric potential to be effective, it is essential to establish an adequate value from which irrigation begins. This threshold value depends on the crop species, its development stage, evaporative conditions and soil texture [27].

Many of the studies carried out for a specific species show a wide range of threshold values of soil matric potential, which suggests, as pointed out by Thompson et al. [28] the influence of site-specific factors. Therefore, it is very important to establish the appropriate value of threshold values of soil matric potential for each crop and specific development condition that optimize production and efficiency in the use of water and nutrients [4,29,30].

The pepper is one of the most important horticultural crops (*Capsicum anuum* L.) for greenhouse production. Specifically, in Spain, its production is concentrated on the Mediterranean coast of Andalusia, highlighting Almería as the main growing area with more than 11,000 ha and an annual production of 845,595 tons [31].

The objective of this work was to determine the influence of the fertigation activation command with electrotensiometers and the characteristics of the greenhouse on the water and nutrients productivity in a pepper crop.

## 2. Materials and Methods

### 2.1. Experimental Site

The trial was carried out at the IFAPA La Mojonera Center, Almería, in two multitunnel greenhouses with a semi-elliptic curved roof of the same surface (900 m^2^) and orientation (east–west), with a passive climate, with a metal structure, plastic polyethylene cover and windows lateral and zenithal. In both greenhouses, the cultivation system was soil with addition of layer of sand about 5 cm, characteristic of intensive horticultural crops in the Southeast of the Peninsula [32].

### 2.2. Experimental Design and Cropping Systems

**Figure 1:**
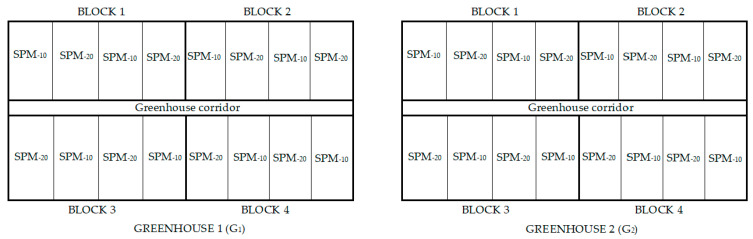
Experimental design.

The experimental design used balanced incomplete block 2 by 2 factorial design with eight replicates (four per greenhouse), resulting in the combination of the two factors studied in the four treatments tested. Each greenhouse was divided into 4 blocks and in each one of them the treatments were placed randomly distributed, resulting in 16 experimental plots per greenhouse (Figure 1). A single plot measured 12 m by 4 m.

The factors studied were: SMP and greenhouse (G).

Two levels of SMP have been established, SMP_−10_: Automatic activation of irrigation through an electronic tensiometer when the level of SMP was −10 kPa, and SMP_−20_: Automatic activation of irrigation through an electronic tensiometer when the level of SMP was −20 kPa. The timing of irrigation was constant throughout the cycle (being 30 min) and the same for two levels of SMP. The frequency of irrigation depended on the value of the SMP established in each treatment. In a previous experiment, we established three levels of soil matric potential −10 kPa, −20 kPa and −30 kPa, as well as a fourth treatment that received the irrigation calculated with the irrigation scheduling based on crop evapotranspiration. Under the development conditions, the soil matric tension threshold of −20 kPa was the one that had shown the best results considering the water and nutrient productivity criteria, but −10 kPa was the highest fruit yield [33]. For this reason, in this experiment we have studied these two levels.

The total volumes of irrigation applied were different in each treatment, since irrigation was managed by the threshold level of soil matric potential established for each treatment. What remained constant in all treatments was the irrigation activation period, which was from 10:00 a.m. to 6:00 p.m. in winter and from 8:00 a.m. to 8:00 p.m. in spring. A 2 h pause was also established after each irrigation to ensure the correct response for the acquisition of tensiometric data.

**Figure 2:**
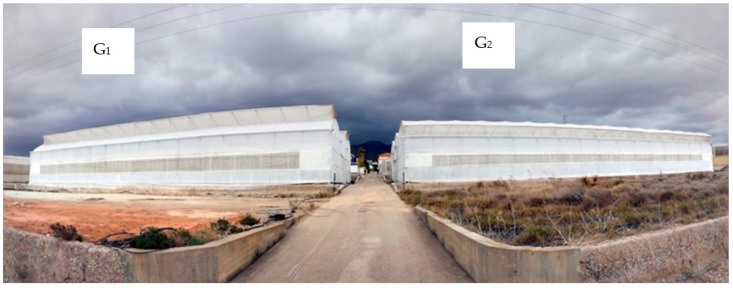
Greenhouse studied.

Two different greenhouses have been studied: G_1_ and G_2_. The differences between the greenhouses were greenhouse height, size and the type of lateral and zenith windows, and the properties of the soil. Greenhouse G_1_ was more height than G_2_ (4.2 m of the gutter height and the total ridge height was 6 m) and more height lateral windows (1.7 m), and had zenith windows at the top of a quarter arch (Figure 2) and soil with a clay loam texture (Table 1). Greenhouse G_2_ had zenith windows at the top of a half arch with an opening in the gutter (Figure 2), 3.6 m of the height to gutter and the total height to ridge was 5.4 m, height lateral windows of 1.25 m and soil with a clay texture (Table 1).

To determine the physical-chemical parameters in the soil twenty random sample points of each replication were selected to be taken and the samples of soil were mixed to achieve a representative soil sample per replication. The samples of soil were dried in a forced air oven (MEMMERT Model 800) at 50 °C for 48 h and then were gridded and screened (2 mm).

Texture of soils (clay, silt and sand percentages) was determined by Bouyoucos-hydrometer analysis [34]. pH and electrical conductivity (EC) were determined in the saturated extract by pHmeter (model MicropH 2002 Crison, Hach Large Spain S.L.U., Barcelona, Spain) and conductivity meter (model GLP31 Crison, Hach Large Spain S.L.U., Barcelona, Spain), respectively. Sodium adsorption ratio (SAR) was determined in the water extract from saturated soil paste. Organic matter was determined using the Walkley–Black method [35].

The interpretation of the soil-water characteristic curves (SWCC) for each soil are show in Table 2. SWCC describes the amount of water retained in a soil (expressed as volume water content, θv) under equilibrium at a given matric potential. The model used to predict such relationship was Van Genuchten [36]. An SWCC is an important hydraulic property, related to size and connectedness of pore spaces, hence strongly affected by soil texture and structure, and by other constituents, including organic matter. The soil of the G_1_ greenhouse has a higher θv than the soil of the G_2_ greenhouse for the same SMP level (Table 2).

Irrigation was applied by drip irrigation. Pressure-compensating and self-closing drippers were employed (PCJ Dripper—Netafim^®^). The flow rate of the emitters was 3 L h^−1^ and two emitters per square meter were installed (spaced at 0.5 m in the drip line and 1 m between lines).

The concentration of the nutrient solution applied was the same for all treatments and remained constant throughout the cultivation cycle, being in mM: 12 of NO_3_^−^, 1.5 of H_2_PO_4_^−^, 1.5 of SO_4_^2−^, 6.0 of K^+^, 5.0 of Ca^2+^ and 2.0 of Mg^2+^.

Pepper plants (*Capsicum anuum* L. type Lamuyo cv. Mazo) were transplanted on 5 September 2018 with a plant density of 2 plants m^−2^ and the cycle ended on 23 April 2019.

### 2.3. Measurements

Climatic parameters in greenhouses: Air temperature and air relative humidity inside the two greenhouses were measured. The measurements were recorded every 30 min during 24 h a day. Daily mean vapor pressure deficit (VPD) was calculated for each greenhouse.

Soil matric potential was measured in the most representative area of the plant roots. For this, 32 electrotensiometers (Irrometer Co., Inc., Riverside, CA, USA) were installed, 8 per treatment. The electrotensiometers were placed 15 cm deep and 15 cm from the base of the plant. Soil matric tension measurements were automatically recorded by a Red Himarcan^®^ System control device.

Soil drainage volume was determined for each treatment. For this, each greenhouse was equipped with 16 drainage lysimeters (one for treatment and replication) of 1 m^2^ of surface, installed at a depth of 50 cm, discounting the upper layer of sand. The volume of water drained was measured daily throughout the experiment.

Irrigation water and nutrients applied were measured by installing three volumetric water meters, model M120 (Elster, Iberconta S.A., Gipuzkoa, Spain), one for treatment, also was corroborated with the registration number of irrigations performed and the volume applied in each irrigation. The concentration of nutrients (NO_3_^−^, P, K^+^, Ca^2+^ and Mg^2+^) in the applied nutrient solution was analyzed in the laboratory weekly. The samples were collected at the outlet by the dropper, placing a carafe that collected the volume of fertigation applied weekly in each treatment.

Yield of the pepper crop was evaluated by manually harvesting the red fruits. The production of 48 plants was controlled per replication and treatment, resulting in a total of 384 plants per treatment. The harvest period began on 9 January 2019 and ended on 23 April 2019, with a total of 5 harvests. The weight and number of marketable and unmarketable fruits were counted. The marketable fruits were separated by size. The average weight of the fruits by size was determined.

Vegetative growth of pepper plants was determined by measuring the dry biomass and the percentage of dry matter of the aerial part of the plant. Crop harvest index (HI, g g^−1^) was calculated as the ratio between generative dry biomass and total shoot dry biomass. For this, whole plants (leave, stem and fruit), excluding roots, were collected from each experimental plot (six plants per repetition) at the end of the experiment and 20 fruits per experimental plot were also randomly selected in each harvest. The fresh samples were weighed and dried at 70 °C to constant weight and the dry weight was determined.

Productivity of water and nutrients was calculated. There are numerous authors who have defined the productivity of water or the efficiency of water use [37,38,39,40,41,42]. We define the productivity the following way:

1. Water productivity (WP); Y/W.
2. Nitrogen productivity (NP); Y/Nc.
3. Phosphorus productivity (PP); Y/P.
4. Potassium productivity (KP); Y/K.
5. Magnesium productivity (MgP); Y/Mg.

where:

Y is fruit marketable yield (kg m^−2^).
W is water applied (m^3^ m^−2^).
N is nitrogen applied (kg m^−2^).
P is phosphorus applied (kg m^−2^).
P is potassium applied (kg m^−2^).
Ca is calcium applied (kg m^−2^).
Mg is magnesium applied (kg m^−2^).

### 2.4. Statistical Analysis

An analysis of variance (ANOVA) was performed for a 2 by 2 factorial balanced incomplete block design with eight repetitions. To identify the significant factors (SMP and G) and the interactions between the factors, a multifactorial ANOVA was performed. When the ANOVA was significant, an least significant difference LSD test (*p* ≤ 0.05) was performed to identify statistically significant differences between the means. To obtain a normal distribution, the percentage data were transformed with an inverse sign √. The statistical software used was Statgraphics 18.

## 3. Results

### 3.1. Climatic Parameters

**Figure 3:**
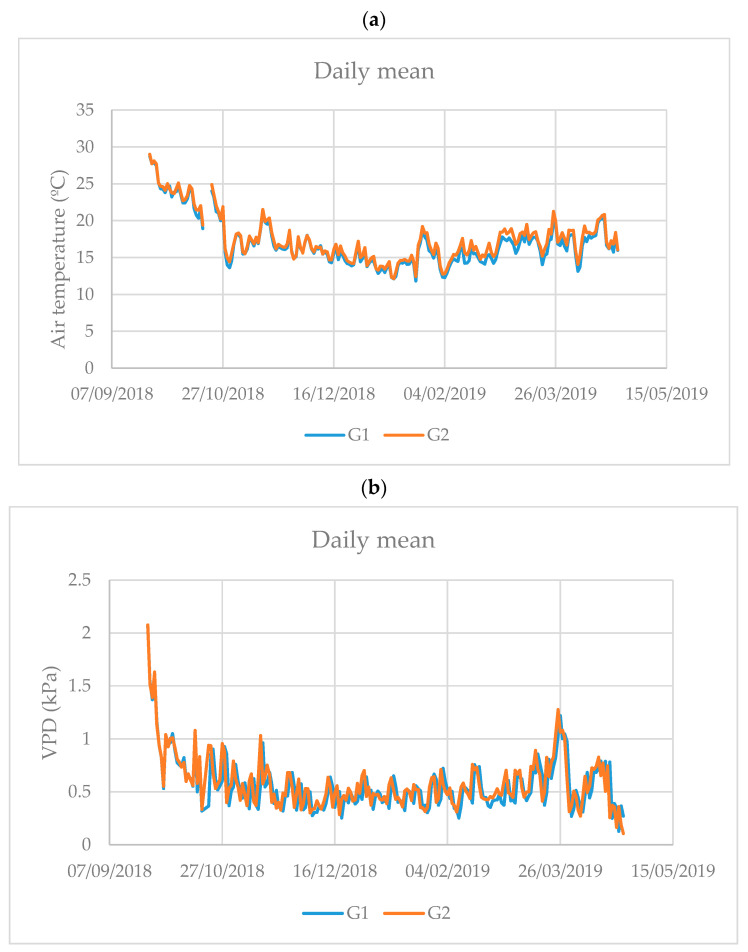
Daily mean air temperature (**a**) and daily mean air vapor pressure deficit (VPD) (**b**) inside of the two greenhouse during the growing seasons.

The average daily air temperature inside the greenhouses fluctuated throughout the growing cycle, being higher at the beginning of the cycle with average values close to 30 °C and with minimum values in the winter months, the lowest recorded average being 12 °C (Figure 3a). During most of the growing cycle the average daily temperature was around 15 °C. It is noteworthy that in the two greenhouses (G_1_ and G_2_) the air temperature was very similar (Figure 3a).

The daily mean VPD also showed fructifications throughout the crop cycle (Figure 3b). At the beginning of the cycle it was high, reaching values of 2 kPa (September 24), although, during most of the growing cycle it was around 0.5 kPa. As with air temperature, the VPD it was similar in the two greenhouses (Figure 3b).

### 3.2. Soil Matric Potential (SMP)

**Figure 4:**
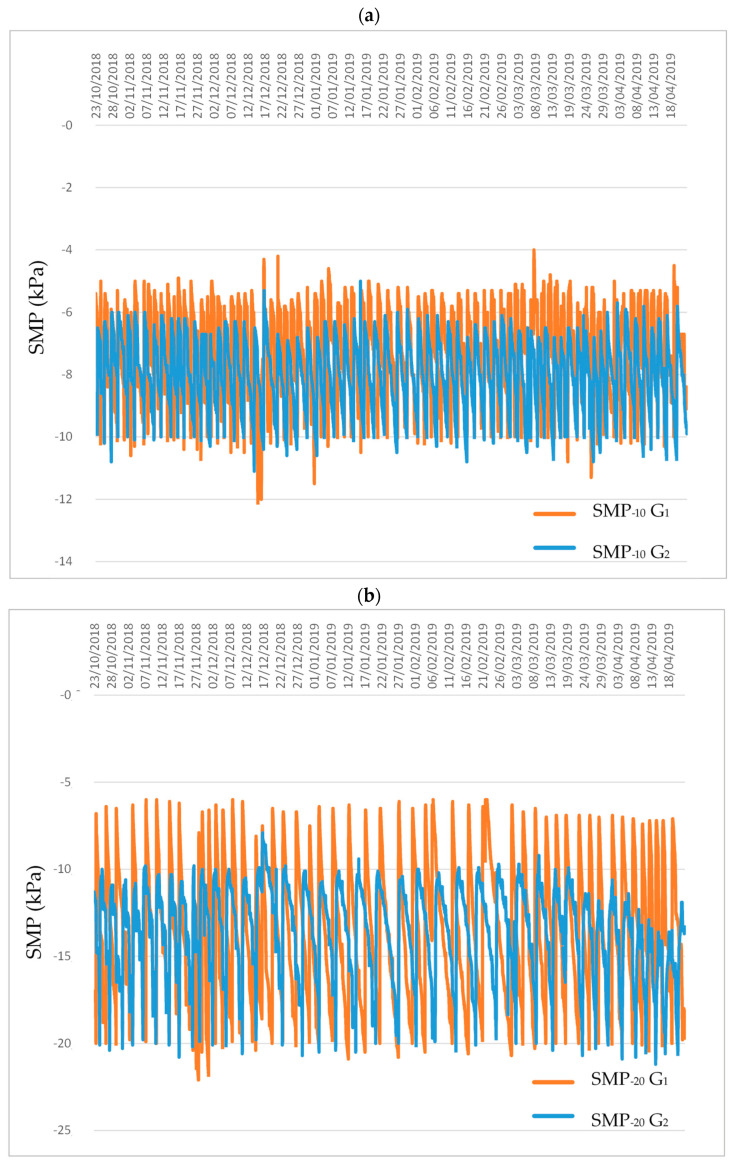
Soil matric potential variations for treatments in the growing seasons. Treatments SMP_−10_ G_1_ and SMP_−10_ G_2_ (**a**) represent controlling soil matric potential thresholds of −10 kPa in the differents greenhouse studied, and treatments SMP_−20_ G_1_ and SMP_−20_ G_2_ (**b**) represent controlling soil matric potential thresholds of −20 kPa in the differents greenhouse studied.

The fertigation treatments began on 23 October 2018. Figure 4 shows the matric potential values of the soil for the established treatments (SMP_−10_ and SMP_−20_) and for each greenhouse. The threshold values of soil matric potential established for each treatment were exactly the setpoints established for each treatment, since the activation of irrigation was established with the reading values of the tensiometers.

Before starting the fertigation treatments, soil matric potential was similar for all treatments and was close to −10 kPa.

Although the same amount of water was applied to each irrigation in both greenhouses, in greenhouse G_1_ the range of SMP was wider than in greenhouse G_2_. The SMP values for fertigation activation at −10 kPa were between −5 and −10 kPa for G_1_, however, they were between −6 and −10 kPa for G_2_. The same was registered with the SMP values for fertigation activation at −20 kPa, registering a greater range (from −6 to −20 kPa) in G_1_ and a smaller range (from −10 to −20 kPa) in G_2_.

### 3.3. Irrigation Water and Nutrients Applied and Drainage Volume

Water and nutrients applied in each level and factor is shown in Table 3. The irrigation activation depending on the level of soil matric potential had an influence on the volume of water applied to the pepper crop. Activation of fertigation at −10 kPa increased the volume of water and applied nutrients by 28% (from 237 to 303 L m^−2^) (Table 3). The greenhouse also affected the volume of water and nutrients applied. G_1_ applied 13% more water and nutrients than G_2_. There was no interaction between the factors studied (Table 3).

To calculate the ETc, the irrigation software PrHo v 2.0© 2008 was used and the climatic data corresponding to the average climatic year was the historical series of the Cajamar Foundation (https://www.fundacioncajamar.es/es/comun/). The aforementioned software calculates the reference evapotranspiration (ETo) using a simplified version of the FAO (Food and Agriculture Organization of the United Nations)-radiation model [8] and adjusts the culture coefficient using the Kc-TTA (crop coefficient -accumulated thermal time) model [6]. For this cycle of greenhouse pepper cultivation, the ETc was 301 L m^−2^.

It is remarkable that for the SMP factor, the level −10 (−10 kPa) registered a water consumption very similar to the ETc calculated (303 vs. 301 L m^−2^).

No drainage was obtained in any of the treatments, regardless of the threshold of soil matric potential and greenhouse (Table 3). These results indicate that the volumes applied by the crop in the different treatments were not in excess at any moment of the cycle, achieving that 100% of the applied water stayed in the profile of the soil used by the roots of the crop.

### 3.4. Yield

The fertigation activation threshold had a significant effect on the total commercial production of pepper, the treatments that had a higher level of soil moisture in the soil (SMP_−10_) obtained higher productions than those with a lower level of soil moisture (SMP_−20_). The average marketable yield in SMP_−10_ was 6.87 kg m^−2^ compared to 5.88 kg m^−2^ in SMP_−20_ (Table 4). When analyzing the marketable yield by size, it was found that these differences in total marketable yield were associated with a higher yield and number of fruits of size I in the SMP_−10_ level, showing no significant differences in yield and number of fruits of size II. The fruits of size I presented an average weight of 390 to 400 g and the fruits classified as size II an average weight of 200 g, without significant differences between levels. Most of the marketable yield corresponded to fruits of size I, representing 91% for SMP_−10_ and 88% for SMP_−20_. Unmarketable fruits represented a very low percentage with respect to commercial production, being 4% for the SMP_−10_ level and 2.7% for the SMP_−20_ level with significant differences between them.

The greenhouse factor also influenced marketable yield, G_1_ was more productive than G_2_ (6.72 vs. 6.04 kg m^−2^). The increase in total marketable yield was associated with a higher production and fruits number of size I. The average weight of the fruits within each size was not modified by the greenhouse factor. The percentage of unmarketable yield was higher in G_1_, although it represented only 4.61% with respect to total yield.

No interaction between factors was registered.

**Figure 5:**
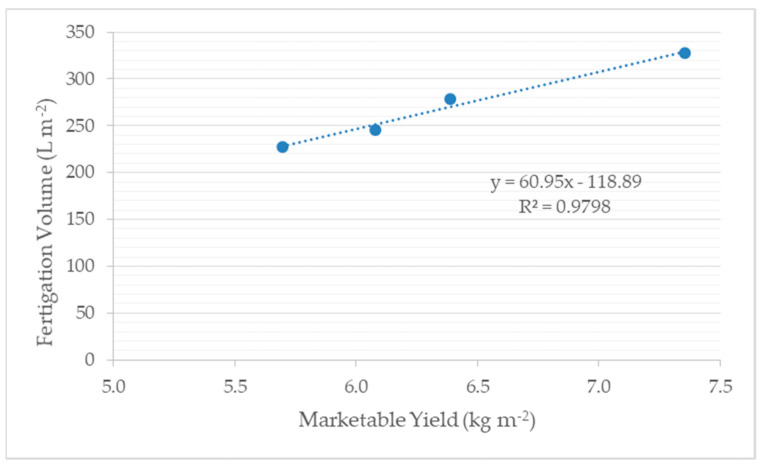
Linear relationship between total marketable yield and fertigation volume applied.

The total marketable yield was very well correlated with fertigation volume applied (Figure 5) with a high determination coefficient (R^2^ = 0.9798) and this relationship was also regressed as follows:

Fertigation volume (L m^−2^) = 60.95 * marketable yield (kg m^−2^) −118.89

### 3.5. Total Dry Biomass

Dry matter content in plant tissue, shoot biomass (dry matter) and its partitioning at the end of the cycle are shown in Table 5. Vegetative growth of pepper plants was influenced by the threshold of soil matric potential established. Total shoot biomass was reduced with decreasing soil matric potential. This reduction was due to a statistically significant reduction in the biomass of stem, leaf and fruit registered at the SMP_−20_ level. The reduction in total dry biomass was estimated at 25% (Table 5). This reduction in biomass could be the main cause of the differences recorded in water consumption between the different treatments.

All treatments recorded a similar HI (Table 5). This indicates that the relationship between the vegetative part (leaf + stem) and the generative part (fruit) did not vary as a function of the matric potential of the soil, and neither as a function of the greenhouse.

### 3.6. Water and Nutrients Productivity

Neither of the two factors studied (SMP and G) affected water and nutrient productivity by pepper crop (Table 6). The WP ranged between 23 and 25 kg m^−3^. The productivity of nutrients varied as a function of the nutrient, ranging the values for NP between 1.9 and 2.1 kg g^−1^, for PP between 15.3 and 16.7 kg g^−1^, for KP between 3.5 and 3.9 kg g^−1^, for CaP between 5.1 and 5.6 kg g^−1^ and for MgP between 15.3 and 16.7 kg g^−1^.

## 4. Discussion

### 4.1. Soil Matric Potential Effect

To maintain a SMP closer to zero (higher level of soil moisture and therefore greater water availability) it is necessary to apply a greater volume of irrigation water, as collected in numerous investigations [4,29,30,43,44,45] and it is corroborated with the results obtained in this experiment.

To maintain the SMP threshold at −10 kPa during the sweet pepper crop cycle, the total volume of irrigation water needed was 303 Lm^−2^. This volume was very similar to the ETc estimated using the methodology proposed by Fernandez et al. [6] with average climate data.

However, to maintain the SMP threshold at −20 kPa throughout the cycle, the necessary volume was 22% lower (237 Lm^−2^). By reducing the volume of water applied, the nutrients applied in fertigation were also proportionally reduced, factors which limited the yield of the crop under our growing conditions [46]. As there was no drainage and therefore no nutrient leachate with any of the soil matric potential thresholds studied, we can say that the volumes of water supplied were entirely used by the crop.

The greater availability of water and nutrients obtained when fertigation is activated at a SMP threshold of −10 kPa increased the yield and number of fruits of the sweet pepper crop with respect to the activation of fertigation at a SMP threshold of −20 kPa, as a consequence of a greater yield and number of fruits of greater size. Different authors have studied the effect of soil matric potential on productivity in different crops and production systems [36,37,38,39,40]. Coinciding with what was observed in this experiment, numerous authors have observed a relationship between SMP and crop yield. The results obtained in this work coincide with those of other trials carried out on the same crop (sweet pepper) and under similar development conditions, in a greenhouse and soil with a layer of sand provided [47,48]. The results were also similar to those obtained in other greenhouse horticultural crops such as zucchini (also in soil with a surface layer of sand) [4,49] confirming that an irrigation activation threshold closer to saturation increases fruit production. Even so, the magnitude of importance varies according to the crop, so that in previous trials carried out in zucchini crop [4] we determined that a modification in the threshold of the soil matric potential from −10 to −25 kPa produces a slight significant reduction in marketable yield, estimated at 10%. However, in this experiment on sweet pepper cultivation, a smaller variation of the soil matric potential (from −10 to −20 kPa) produced a greater decrease in commercial production (14%, from 6.87 kg m^−2^ to 5.88 kg m^−2^).

Specifically, Létourneau et al. [30] managed to improve strawberry crop yields grown in the open field by 6.2% when they worked with a soil matric potential of −15 kPa instead of −20 kPa. Similarly, for tomato crops grown in the open field, Zheng et al. [45] obtained a reduction in production between 23.0% and 27.7% depending on the season, by reducing the matric potential of the soil from −10 kPa to −50 kPa and Buttaro et al. [29] established that the greenhouse tomato irrigated at the potential of −40 kPa showed a 40% lower yield (mainly due to the smaller size of the fruit) compared to the plants watered at −10 kPa.

As was observed in crop yield, the development of the plant was also affected by the irrigation activation SMP threshold, obtaining higher aerial dry biomass in the treatments in which −10 kPa was established compared with −20 kPa. This higher dry biomass was associated with a higher production of fresh biomass, higher vegetative development, since the percentage of dry matter in the tissues was not altered by the SMP values studied. HI was high (between 63% and 65%) and was not affected by the levels of SMP studied, conserving the proportion of fruit and vegetative part. Coinciding with numerous investigations [4,29,43,44], deficit irrigation produces as a reduction in vegetative growth.

The irrigation activation threshold of −10 kPa maintained the productivity of water and nutrients in agronomic terms. Water productivity in agronomic terms was around 24 kg of commercial pepper fruit per m3 of applied fertigation, being similar to that obtained in previous trials under similar development conditions [47,48]. Numerous studies show how increasing the volume of water and nutrients applied reduces their productivity, although fruit production increases [4,29,30,43,49]. However, in this trial, productivity has been preserved and fruit production has increased, and it can be concluded that, under the trial development conditions, the −10 kPa treatment has been the best.

### 4.2. Greenhouse Effect

The greenhouse factor affected the volume of fertigation applied. Growing in the G_1_ greenhouse required 13% more fertigation than the G_2_ greenhouse. These differences between the greenhouses in the volume of fertigation demanded could be the consequence of different environmental conditions between greenhouses (caused by different ventilations) and of the different types of soil (different textures and moisture retention curves). Sánchez–Guerrero et al. [23] and Stanghellini et al. [24] found that within the greenhouse cultivation system there are several factors such as: Greenhouse structure, ventilation and soil type. that determine and modify the consumption of water and fertilizers by modifying the environmental conditions in which the crops develop and the level of development of said crop. However, in this trial, the results of the climatic parameters inside the greenhouse were similar in both greenhouses (Figure 3). Although the height of the greenhouses and the ventilation surface were different, neither the temperature nor the VPD were modified, so these differences in the consumption of fertigation volume must have been associated with the soil. The variation of the soil matric potential was different in each of the greenhouses (Figure 4), the soil of the greenhouse G_1_ showed more variation of SMP than the soil of G_2_ with the same depth of applied water (3 L m^−2^ corresponding 30 min of irrigation time). The G_1_ soil presented lower levels of SMP after irrigation associated with the retention curve characteristic of each type of soil (Figure 4). The SMP values for fertigation activation at −10 kPa were between −5 and −10 kPa for G_1_, however, they were between −6 and −10 kPa for G_2_. The same was recorded with the SMP values for the activation of fertigation at −20 kPa, registering a higher range (−6 to −20 kPa) in G_1_ and a lower range (−10 to −20 kPa) in G_2_.

The greenhouse also affected the commercial yield, the greenhouse G_1_ had 11% more production than the greenhouse G_2_ (6.7 vs. 6.0 kg m^−2^). This percentage was similar to the increase in fertigation demanded in this greenhouse. The higher demand for fertigation carried out in the G_1_ greenhouse was possibly associated with the greater development of the crop in this greenhouse, not only the commercial production was higher, but also the dry biomass of the crop (of leaves, stem and fruit) was higher associated with higher fresh biomass, since the percentage of dry matter in the tissue was the same for the cultivation in the two greenhouses studied. This higher growth was possibly associated with the more favorable level of average matric potential recorded in the soil of the G_1_ greenhouse, which has already been commented on previously, since the climatic conditions recorded in the greenhouses were similar. The higher fruit production and crop development when irrigation is activated at a matric potential closer to zero has been widely corroborated by different authors and in different crops [4,29,30,45,47,48]. The highlight from the results obtained in this experiment was that no only SMP threshold at which irrigation was activated had an influence, the SMP reached after finishing the irrigation also had an influence on the production and growth of the crop.

The two greenhouses registered high water and nutrient productivities without significant differences between them. Water productivity in agronomic terms was around 24 kg of commercial pepper fruit per m^3^ of fertigation applied for the two greenhouses, being similar to that obtained in previous trials under similar development conditions [46,47].

## 5. Conclusions

The results obtained in this work showed the great importance of establishing an optimum level of SMP threshold value to automate fertigation with electrotensiometers in greenhouse pepper crops. Under the development conditions of the experiment, the activation threshold of −10 kPa increased fruit production as well as the biomass and conserved water and nutrients productivity with respect to the activation threshold of −20 kPa in both greenhouses.

The greenhouse factor also had a significant influence on fruit production, the crop in greenhouse G_1_ obtained higher yields and vegetative development, also requiring a greater volume of fertigation than the crop developed in the greenhouse G_2_ for the same activation threshold. This better response was associated with a more favorable average matric potential recorded in the soil of this greenhouse, since the matric potential threshold after irrigation was closer to zero. Therefore, it was shown that not only the SMP threshold used by fertigation activation had an influence, but also the lowest SMP threshold obtained after fertigation, confirming that it is necessary to establish depth irrigation that achieve a SMP closer to zero to achieve increased fruit yield and crop growth. Therefore, it is essential to know the soil retention curve to adapt the irrigation endowment and reach a potential close to zero without producing losses due to deep filtration (drainage).

The automation of fertigation with electrotensiometers allowed applying the volume of fertigation demanded by the crop, which was different according to the soil conditions of each greenhouse, and was also different depending on the threshold value of the soil matric potential established for activation and also allowed to eliminate water losses by drainage and therefore the leaching of nutrients.

## Figures and Tables

**Table 1:** Physical and chemical properties of the soil. Electrical conductivity (EC), pH and sodium adsorption ratio (SAR) were determined in the water extract from saturated soil paste. Organic matter (OM) and exchangeable cations.

									Exchangeable Cations			
	Texture	Sand	Silt	Clay	EC	pH	SAR ^a^	OM ^b^	Na	K	Ca	Mg
			%		dS m^−1^			%	meq/100 g			
G_1_	clay loam	22	46	32	4.42	9.0	2.1	0.41	0.0	2.6	3.7	2.1
G_2_	clay	34	18	48	2.97	8.7	2.8	0.78	0.1	4.3	5.4	4.2

^a^ Sodium Adsorption Ratio, ^b^ Organic Matter.

**Table 2:** Interpretation of the soil-water characteristic curves (SWCC) for each soil.

SMP (−kPa)	θv (%)	
	G_1_	G_2_
0	25.46	22.88
10	23.13	16.56
20	21.64	14.61
30	20.59	13.50
33	20.35	13.26
40	19.86	12.79
50	19.24	12.23
100	17.39	10.66
500	13.51	7.71
1000	12.09	6.71
1500	11.31	6.17

**Table 3:** Total water and nutrients applied and drainage volume.

		Water	N	P	K	Ca	Mg	Drainage
		L m^−2^			g m^−2^			L m^−2^
SMP		*	*	*	*	*	*	ns
	−10	303.11	3.64	0.45	1.97	1.36	0.45	0.0
	−20	236.76	2.84	0.36	1.54	1.07	0.36	0.0
G		*	*	*	*	*	*	ns
	1	286.49	3.44	0.43	1.86	1.29	0.43	0.0
	2	253.39	3.04	0.38	1.65	1.14	0.38	0.0
Interaction		ns	ns	ns	ns	ns	ns	ns

* significance for *p* ≤ 0.05; ns, no significance.

**Table 4:** Total marketable yield, marketable by sizes and unmarketable pepper yield.

		Total Marketable	Marketable by Size						Unmarketable
			Size I (>250 g)			Size II (<250 g)			
		kg m^−2^	kg m^−2^	Fruits m^−2^	Fruits Weight (kg)	kg m^−2^	Fruits m^−2^	Fruits Weight (kg)	%
SMP		*	*	*	ns	ns	ns	ns	*
	−10	6.87	6.18	15.73	0.39	0.69	3.50	0.20	4.0
	−20	5.89	5.25	13.07	0.40	0.63	3.20	0.20	2.7
G		*	*	*	ns	ns	ns	ns	*
	1	6.72	6.07	15.43	0.40	0.64	3.37	0.19	4.61
	2	6.04	5.36	13.38	0.40	0.68	3.33	0.20	2.09
Interaction		ns	ns	ns	ns	ns	ns	ns	ns

* significance for *p* ≤ 0.05; ns, no significance.

**Table 5:** Stem, leaf and fruit dry matter. Vegetative, generative and total shoot biomass, and crop harvest index (HI).

		Dry Matter Content (%)			Shoot Biomass (g m^−2^)				HI (g g^−1^)
					Vegetative		Generative	Total	
		Stem	Leaf	Fruit	Stem	Leaf	Fruit		
SMP		ns	ns	ns	*	*	*	*	ns
	−10	20.7	17.2	9.3	220	170	664	1054	0.63
	−20	21.3	18.0	9.4	174	140	576	890	0.65
G		ns	ns	ns	*	*	*	*	ns
	1	21.0	17.5	9.3	209	166	650	1025	0.63
	2	21.0	17.7	9.4	184	144	590	918	0.64
Interaction		ns	ns	ns	ns	ns	ns	ns	ns

* significance for *p* ≤ 0.05; ns, no significance.

**Table 6:** Water productivity (WP) (kg of commercial fruit per m^3^ of water applied) and nutrients productivity (kg of commercial fruit per kg nutrient applied). NP: Nitrogen productivity, PP: Phosphorus productivity; KP: Potassium productivity, CaP: Calcium productivity and MgP: Magnesium productivity.

		WP	NP	PP	KP	CaP	MgP
		(kg m^−3^)			(kg g^−1^)		
SMP		ns	ns	ns	ns	ns	ns
	−10	22.87	1.91	15.25	3.52	5.08	15.25
	−20	24.76	2.09	16.73	3.86	5.58	16.73
G		ns	ns	ns	ns	ns	ns
	1	23.40	1.98	15.82	3.65	5.27	15.82
	2	24.23	2.02	16.15	3.73	5.38	16.15
Interaction		ns	ns	ns	ns	ns	ns

ns, no significance.

## Abbreviations

The following abbreviations are used in this manuscript:

SMP	Soil Matric Potential
G	Greenhouse
SWCC	Soil-Water Characteristic Curves
VPD	Vapor pressure deficit
WP	Water Productivity
NP	Nitrogen Productivity
PP	Phosphorus Productivity
KP	Potassium Productivity
CaP	Calcium Productivity
MgP	Magnesium Productivity
HI	Harvest Index
ETc	Evapotranspiration of crop
